# Progress in Medicine

**Published:** 1896-08-01

**Authors:** 


### Progress in Medicine.

### DISEASES OF THE STOMACH.

**Diagnosis.**—In the diagnosis of various affections where it is inconvenient to pass a tube, Boardman Reed[1] finds definite knowledge can be obtained by percussion and clapotement; the former being the most reliable. The examination should be carried out six hours after a meal, or before breakfast. If clapotement can then be heard there is sufficient motor power, and the boundaries should be determined by percussion, first in the recumbent and then in the upright position. The patient should then drink an eighth of a litre of water, and the examination is repeated; after which another eighth should be given, and again the same tests are employed. If clapotement is heard with the smaller amount the motor power is doubtful, and if the dulness extends farther downwards with successive draughts of water an atonic condition is present. He distinguishes thus: (1) The normal stomach, where no splash is heard till the stomach is partly full, and often not even then. The position of the boundaries is normal. (2) The atonic stomach. A splash may be heard four to six hours after a full meal, or a little water will develop it decidedly. Percussion will show delayed emptying of the organ. (3) Megalogastria, or enlarged stomach with good motor power. The lower border may not be higher than the umbilicus, but there is no splash when fasting. (4) Dilatation or gastrectasia. A splash is heard when fasting. Percussion shows enlargement and delay in emptying. (5) Gastroptosis, or downward displacement of a normal stomach. The splash is rather more easily obtained; percussion shows that the upper and lower boundaries are both lower. (6) Gastroptosis combined with an enlarged but not atonic stomach; (7) gastroptosis and dilatation; (8) pyloroptosis, where the pyloric end is displaced downwards, and the area of dulness is normal in size but vertical in position. The splash is heard far below the normal, even when the motor power is quite normal. (9) Pyloroptosis with dilatation. The splash is found when fasting, and the area of the pyloric end is widened. Boas[2] employs a soft bougie with a sponge attached, which is made to rotate as in Turck's instrument. The movements can be felt through the abdominal walls in most patients, and the whole area of the greater curvature can thus be mapped out. The view is gaining ground that the stomach has comparatively little importance in digestion, but is engaged in rendering materials harmless for the intestines by maceration, heating, and disinfection. The fundus is primarily a receptacle for food, but the motor power of the pyloric portion is the most important function of the stomach. Schüle[3] finds that the fasting stomach when the patient awakes from sleep contains from 2 to 23 cub. cm. of fluid, acid in reaction, and containing gastric juice, but free H.Cl. was found only seven times out of 34. He regards a hyper-acid or an alkaline secretion as pathological, but the gastric juice found normally is due to the stimulus of the saliva swallowed,

**Ulcer.**—Paul Cornet[4] advises when hæmorrhage is present, that not even ice should be given by the mouth; ergot should be injected in the neighbourhood of the stomach, and nutrient enemata employed. A blister or ice compress and opiates may be useful. Some days after hæmorrhage has ceased warm liquid food may be given, such as milk, beef-tea, and egg emulsions. In the second week hot compresses are constantly employed, and Carlsbad salts night and morning with the previous diet. The salts are continued for six weeks, the diet being gradually relaxed, but raw fruits, highly-seasoned food, and hot or iced drinks must be avoided for a long period. If there has been no hæmorrhage, various plans of treatment give good results. Thus Cornet refers to Donkin's method of rectal feeding for two or three weeks and hot compresses on the epigastrium; also to Pleiner's use of large quantities of bismuth after lavage of the stomach daily, and to the administration of belladonna and codeia by Boas.

**Cancer.**—J. A. Wesener[5] has tested and recommends Boas' estimation of lactic acid as acetic aldehyde, and denies that it occurs normally or in simple dilatation. Dreschfield[6] writes on early diagnosis where no tumour can be felt. In elderly people there may be no emaciation, and at first only diminished appetite and distaste for nitrogenous food. Pain and occasional vomiting after food then appears, and the epigastrium is painful. There is soon to be found absence of H.Cl., and the presence of lactic acid. In patients from forty-five to fifty-five, emaciation, pain, and loss of appetite occur at an earlier stage. The disease must be diagnosed from chronic gastric catarrh, where there is little loss of flesh, much stringy mucus vomited, and relief under careful diet: from atonic dyspepsia and the dyspepsia of neurasthenia, where there is often displacement of some of the viscera, such as a floating kidney and, lastly, from gastric ulcer. Mintz[7] denies the assertion of Ewald that the secretions of the stomach cease when cancer attacks the œsophagus, and describes three cases in which its activity was maintained. A case of diffuse phlegmonous gastritis is described by Kelynack.[8] The patient had suffered for five years from dysphagia caused by a fibrous stricture of the œsophagus, and was under treatment for that when the temperature began to rise and fall in an unaccountable manner. Some slight abdominal pain occurred, and in ten days' time he became collapsed and died. The stomach was found to be of a yellowish-white colour, the walls very much thickened and infiltrated everywhere with pus, which oozed out on pressure. There was, in addition, an abscess connected with the stricture, but no ulceration or new growth in the stomach. Another case of this rare disease is described by Leith,[9] of Edinburgh. The duration of the illness was one week, and was marked by vomiting and pain. Peritonitis set in, and laparotomy was performed in vain. The stomach walls were greatly thickened, the submucosa being in some parts half an inch in thickness. Immense numbers of micrococci were present. The cause of the disease was not ascertained. It has sometimes been found to be a primary condition as here, and at other times it is secondary to pyæmia. Bondot[10] records two instances of hæmatemesis, which closely simulated gastric ulcer, and which turned out to be associated with inflammation of the biliary passages. The disease commenced with fever, enlarged spleen, and heart murmur; there were no physical signs of liver disease and no jaundice, nor any ulcer of the stomach or varix of the œsophageal veins.

**Functional Diseases.**—Linossier[11] considers that the dose of bicarbonate of soda given for deficient secretion of H.Cl. should be carefully proportioned to the deficiency, and vary from eight to thirty grains. Too small a dose will fail in effect, and too large a one will neutralize the acid evolved. Small doses should be given fifteen minutes, and large ones sixty minutes, before a meal; or, rather, at such time that the meal coincides with the feeling of hunger which follows after a dose. The immediate effect is to produce a feeling of satiety, and shortly after a desire for food. In hyperchlorydria small repeated doses may be given every half-hour during digestion, beginning before the usual commencement of the pain, and continuing until digestion is finished. Each dose of fifteen to thirty grains is too small to cause saturation and, therefore, stimulation. It may be combined with a little magnesia and bismuth, and increased or decreased after some days if necessary. Irrigation with nitrate of silver in cases of chronic catarrh with deficient H.Cl. has been tried with great success by Reale.[11] He commenced with three and three-quarter grains in five drachms of water, after which the stomach was washed out with a 5 per cent. salt solution. He found that vomiting was stopped, and the H.Cl. was increased. The motor power of the stomach and the weight of the patients were increased. Kellog[12] examined the bacteria of the stomach in 377 cases. Of these examinations 191 proved to be absolutely sterile, while in only 102 were there bacteria to the amount of more than 200 to the cubic centimetre at the end of the first hour of digestion.

This certainly shows strong proof of the power of the stomach to destroy microbes. Among the completely aseptic stomachs hypopepsia as well as hyperpepsia occurred, and in some free H.Cl. was absent altogether. Chittenden remarks that free H.Cl. is not absolutely necessary for vigorous digestion of the proteid matter in the stomach. This may be carried out by combined acid. Einhorn[13] reports on the use of Tœpfer's test, dimethyl-amido-azobenzol, for free H.Cl., which is twice as delicate as Gunsburg's. He finds, however, that it fails if lactic acid be present, even to the amount of a tenth per cent., otherwise it is a useful quantitative test. H. C. Crouch[14] refers to the importance of distinguishing true dilatation with stagnation of food through an entire night or long interval of fasting from a simply enlarged stomach with slow digestion, which does empty itself after an interval; and still more from the simply enlarged stomach. Stagnation is the important factor, and this may or may not be accompanied by enlargement. Besides the use of lavage, he emphasises the value of early surgical interference in suitable cases. A fatal case of tetany occurring in dilatation of the stomach is recorded by R. Hill Brown.[15] The patient had long suffered from chronic gastric catarrh, and had had a previous attack of tetany. On its recurrence systematic lavage was used with good effect on the stomach, but renewed convulsions from time to time came on, and at length became fatal. The writer was led to discuss the advisability of lavage in such cases. A useful account of recent theories with respect to motor disturbances of the stomach is given by Kaufman in the *New York Medical Journal* of March 28th, wherein he regards as its most important function that of preparing and passing on the food in small quantities to the intestine for digestion.

W. G. A. Robertson[16] has carried out experiments on the digestion of starch in the stomach, and finds that ptyalin ferment ceases to act very quickly when the normal acid secretions exist, but where the degree of acidity is very feeble, as in some diseases, amylolysis may go on to its full extent. The change ceases when the free H.Cl. reaches ·06 per cent. If proteids are also present this point is reached later, and hence a longer time is afforded for the gastric digestion of starch. There does not appear to be any other agent except ptyalin which is capable of acting on starch in the stomach.

[1] Int. Med. Magaz., March. [2] B.M.J., March 7. [3] Am. J. Med. Sc., April. [4] N.Y. Med. J., April 4. [5] Int. Med. Mag., Feb. [6] Med. Chron., May. [7] B.M.J., May 30. [8] Lanc., March 14. [9] Edin. Med. J., May. [10] Am. J. Med. Sc., March. [11] N.Y. Med. J., May 2. [11] N.Y. Med. J., May. [12] Med. Rec., June 6. [13] N.Y Med. J., May 9. [14] Interl. Med. Mag., May. [15] Lanc., March 21. [16] Ed. Med. J., May.

